# Thyrotoxic Periodic Paralysis: An Unusual Cause of Quadriparesis

**DOI:** 10.7759/cureus.29355

**Published:** 2022-09-20

**Authors:** Nidhi Kaeley, Salva Ameena M S, Silpa S, Anand M Gangdev, Mukund Rajta

**Affiliations:** 1 Emergency Medicine, All India Institute of Medical Sciences, Rishikesh, Rishikesh, IND

**Keywords:** hypokalemia, thyroid storm, quadriparesis, thyrotoxic periodic paralysis, tpp

## Abstract

Thyrotoxic periodic paralysis (TPP) is an uncommon disorder characterized by acute onset of hypokalemia (serum potassium level less than 3.5 mmol/L) and paralysis secondary to thyrotoxicosis. Patients can present with TPP as the first clinical manifestation of thyrotoxicosis. In patients presenting with acute episodes, the presence of hypokalemia and elevated levels of thyroid hormones with low thyroid-stimulating hormone levels (less than 0.35 µIU/mL) are important diagnostic clues.

We report one case of TPP in which the acute onset of paralysis was the first clinical presentation of underlying thyrotoxicosis. After treatment with propranolol and carbimazole, the patient became symptom-free and euthyroid.

## Introduction

Thyrotoxic periodic paralysis (TPP) is a rare complication of hyperthyroidism. Acute onset of severe hypokalemia and profound proximal muscle weakness in patients with thyrotoxicosis are typical of TPP [1]. This condition is most commonly found in Asian men. Although it is usually transient, it can be potentially serious. It is often missed at the first attack due to its low prevalence. The underlying hyperthyroidism is subtle in most cases, which causes difficulty in early diagnosis [2]. In a patient with hypokalemic periodic paralysis, the clinician should not miss the subtle features of hyperthyroidism.

Here, we present the case of a patient who presented to the emergency with episodic weakness, later found to be TPP.

## Case presentation

A 45-year-old female with no known comorbidities and not on any previous medications presented to the emergency department (ED) with complaints of weakness in all four limbs for the past 12 days which started in the lower limb, with distal to proximal progression, and progressed to involve the upper limb in two to three days. The patient had a history of similar complaints eight months back which were resolved without any residual weakness for four to five days. There was no history of fever, vomiting, headache, seizure, or trauma.

The patient was conscious and oriented to time, place, and person on examination. Initial vitals were a pulse rate of 112 beats per minute, blood pressure of 140/90 mmHg, and respiratory rate of 22 breaths per minute. Initial oxygen saturation was 90% at room air which improved to 95% with 3 L oxygen via nasal prongs. The neurological examination revealed the power of 2/5 in proximal muscles and 3/5 in distal muscles in both upper limbs and lower limbs with decreased tendon reflexes and intact sensation. Cerebellar signs and meningeal signs are negative. Other system examinations were within normal limits.

**Figure 1:**
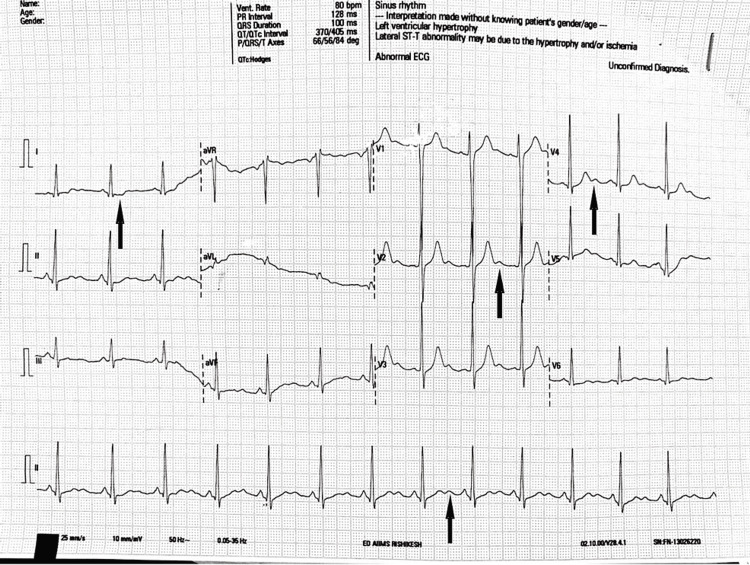
Electrocardiogram of the patient showing features of hypokalemia.

Laboratory examination revealed hypokalemia, as shown in Table 1. Subsequent blood work showed a very low thyroid-stimulating hormone (TSH) with elevated free thyroxine (FT4) and free triiodothyronine (FT3) levels, as shown in Table 1. An electrocardiogram (ECG) showed normal sinus rhythm with no ST elevation and positive U wave (Figure 1). Non-contract computerized tomography of the head was taken which did not detect any anomalies.

**Table 1:** Laboratory investigations. CBC = complete blood count; TLC = total leukocyte count; DLC = differential leukocyte count; SGOT = serum glutamate oxaloacetate transaminase; SGPT = serum glutamate pyruvate transaminase; TSH = thyroid-stimulating hormone; FT3 = free triiodothyronine; FT4 = free thyroxine

Laboratory parameters	Reference range	Day 1	Day 3	Day 5	Day 7
Hemoglobin (g/dL)	11.5–15	11.6	11.2	11.4	11.5
TLC (cells/mm^3^)	4,000–10,000	6,800	8,910	7,840	7,560
DLC (%)		N78 L32	N81 L18	N76 L38	N75 L39
Platelet count (thousand/mm^3^)	150–450	164	168	159	162
Serum potassium (mmol/L)	3.5–5.1	1.98	3.01	3.68	3.91
Serum sodium (mmol/L)	136–146	136	138	142	139
Urea (mg/dL)	10–43	26.7	25.9	-	28.8
Creatinine (mg/dL)	0.5–1	0.9	1.02	-	0.9
Total bilirubin (mg/dL)	0.3–1.2	0.83	-	-	0.79
Direct bilirubin	<0.2	0.12	-	-	0.10
SGOT (U/L)	<35	89	32	-	28
SGPT (U/L)	<35	91	34	-	31
Serum albumin (g/dL)	3.5–5.2	3.62	-	-	3.58
TSH (µIU/mL)	0.35–5.5	0.006	0.08	0.21	0.38
FT3 (pg/mL)	2.3–4.2	12.98	9.71	4.26	3.98
FT4 (ng/mL)	0.89–1.76	6.19	4.19	1.89	1.65

The patient was treated with 40 mEq of potassium chloride at 10 mEq/hour via a peripheral line. During the ED stay, the patient developed respiratory distress and chest tightness. Arterial blood gas analysis showed type two respiratory failure with pCO_2_ of 92 mmHg. Subsequently, the patient was intubated and kept on ventilatory support in volume-assisted control mode.

A provisional diagnosis of TPP with respiratory muscle involvement was made. The patient was started on tablet propranolol 40 mg thrice daily and tablet carbimazole 20 mg twice daily. The patient improved with treatment and was extubated on day two. She was discharged on day eight in stable condition without any residual weakness and is now maintaining follow-up in the endocrinology outpatient department.

## Discussion

Hypokalemic paralysis is characterized by the occurrence of muscle weakness which is episodic and can be caused by gastrointestinal or renal loss of potassium, increased intracellular shift of potassium, or due to rare channelopathies. TPP is common in Asian males and Graves’ disease is the culprit behind most of them [3]. It can be precipitated by stress, strenuous exercise, high-carbohydrate meals, and steroids [2].

The activity of Na+/K+ ATPase is increased in patients with TPP which supports the role of Na+/K+ ATPase in the pathogenesis of TPP. The thyroid hormone stimulates and upregulates the Na+/K+ ATPase which causes increased potassium influx to the cell causing a decreased extracellular level of potassium [4]. During periods of stress and strenuous exercise, the release of catecholamine is also associated with an increase in Na+/K+ ATPase in skeletal muscle [5]. A high-carbohydrate diet leads to an increase in the release of insulin, which has been shown to stimulate Na+/K+ ATPase activity by insulin-response sequence in Na+/K+ ATPase genes [6]. Androgens also play a role in the expression and activity of Na+/K+ ATPase [7]. Some studies have shown that the thyroid hormone upregulates the transcription of the gene encoding kir2.6, a skeletal muscle-specific kir channel associated with TPP [8].

Graves’ disease has been identified as the most common cause of hyperthyroidism in patients presenting with TPP. However, TPP can be triggered by any other cause of hyperthyroidism, even the administration of an excessive amount of exogenous thyroid hormone [9].

Usually, it causes weakness in the proximal muscles of the lower extremities, but with severe potassium depletion, total paralysis including respiratory, bulbar, and cranial musculature can occur. Mortality due to respiratory arrest and arrhythmias is also reported [9,10].

Hypokalemia in TPP occurs because of the shift of potassium into cells and not due to total body potassium depletion. Therefore, correction of the underlying hyperthyroid state is the mainstay of therapy. Non-selective beta-blockers such as propranolol have an important role in early treatment with anti-thyroid drugs or after radioactive iodine treatment when a euthyroid state is not achieved [2]. Correction of hypokalemia with potassium supplementation has been shown to decrease the duration of paralysis and hospital stay [11]. One study compared treatment with intravenous potassium supplementation to oral supplementation and found that the recovery time was less in patients treated with intravenous potassium supplementation than in those who receive oral potassium supplementation [12]. Rebound hyperkalemia is a complication that can occur in patients of TPP who are treated with potassium supplementation. Previous studies are suggestive of a positive correlation between the degree of rebound hyperkalemia and the dose of potassium administered. Patients receiving a total dose of potassium chloride of less than 50 mEq/day rarely develop hyperkalemia [2].

## Conclusions

In patients presenting to emergency with acute-onset quadriparesis, TPP should be considered and distinguished from other causes of acquired quadriparesis, including familial hypokalemic periodic paralysis, Guillain-Barré syndrome, and proximal myelopathy. Early diagnosis and correct treatment will help the clinician to prevent further morbidity and mortality.
